# Supplementary material for: Mining a human transcriptome database for chemical modulators of NRF2
Source: PLoS One. 2020 Sep 28;15(9):e0239367. doi: 10.1371/journal.pone.0239367 (PMC7521735; doi:10.1371/journal.pone.0239367)
Supplement: Attachment — Submitted filename: Response to reviewer comments hNrf2 08_19_20.docx [file pone.0239367.s003.docx]

**Response to second round of reviewer comments**

**For**

**Mining a human transcriptome database for chemical modulators of NRF2**

John P. Rooney^1,2,#a^, Brian Chorley^1^, Steven Hiemstra^3^, Steven Wink^3^, Xuting Wang^4^, Douglas A. Bell^4^, Bob van de Water^3^, and J. Christopher Corton^1*^

^1^Center for Computational Toxicology and Exposure, US-EPA, Research Triangle Park, NC 27711

^2^ORISE, US-EPA, Research Triangle Park, NC 27711

^3^Division of Toxicology, Leiden Academic Center for Drug Research, Leiden University, Leiden, The Netherlands.

^4^ Immunity, Inflammation, and Disease Laboratory, National Institute of Environmental Health Sciences, Research Triangle Park, NC 27709

^#a^Current Address: ILS, Durham, NC

Reviewers' comments:

Reviewer's Responses to Questions

**Comments to the Author**
Reviewer #2: The manuscript is much clearer. Only minor revisions required.
**We thank the reviewer for providing suggestions that helped to make this a better paper.**

OVERALL

The official gene symbol is 'NFE2L2'; but the synonym 'NRF2' has probably been used broadly. To avoid confusion, can you state this at the beginning of the manuscript, and use only one of these two names in the figures and tables? For instance, it is confusing to have in figure 3C a title 'Nrf2 upstream regulators' and then 'NFE2L2' listed as one (but it's actually the same as NRF2)
**We have now made a number of changes suggested by the reviewer. These include stating that Nrf2 is encoded by the NFE2L2 gene in both the abstract and introduction when Nrf2 is first introduced, changing NFE2L2 to Nrf2 in Fig 3C, and adding two changes in Table 1 to indicate that either the Keap1 or NFE2L2 gene are knocked down.**

METHODS

<< The average fold-change across the 7 biosets in which NRF2 was activated had to be > |+/- 1.5-fold|. >> -- is this a log2 (FC)..? Please state explicitly
**We have now changed the sentence to read “The average fold-change across the 7 biosets in which NRF2 was activated had to be ≥ |+/- 1.5-fold| (not Log2(fold-change).”. We hope this suffices.**

<< The p-value is the probability of the overlap between the NRF2 biomarker gene list and the IPA pathway gene list. The smaller the p-value the less likely that the association is random. >> -- can you adopt a more rigorous explanation?

**We removed the last sentence mentioned and added “****Significant reported pathways have q-values < 1E-3.”.**


RESULTS

Building the NRF2 biomarker and assessment of predictive accuracy

In the response to one of the reviewer comments the authors state << First, we did not use the biosets used in making the biomarker in our prediction study. That is we did include the “training set”. >> -- I think the authors meant "we did *not* include the “training set”"; can you make this clear in the results and methods?

**Thanks for picking up this mistake. We have now changed the sentence on line 258 to read “****The biosets used to create the biomarker (essentially the training set) were excluded from the test.” As suggested, we added the same sentence to the Results on line 409.**

Fig 2A: can the authors add labels with the name of the chemicals, or add a table with this information?
**We have now provided this information in Table S1. We now state on line 414 “Information about the biosets used in the test are provided in Table S1.”**

Characterization of the NRF2 biomarker genes.

<< Of the 143 NRF2 biomarker genes, 38 (26.6% ) of these were associated with the NRF2- bound ChIP-Seq loci near gene promoter regions (within 10 Kb) in at least one dataset derived from cell lines treated with NRF2-activating isothiocyanate, SFN, or have constitutively active NRF2 >> -- can you report what's the background rate, when considering all genes?

**We have now reported the background rate in the Results. The text now reads: “Of the 143 NRF2 biomarker genes, 38 (26.6% ) of these were associated with the NRF2-bound ChIP-Seq loci near gene promoter regions (within 10 Kb) in at least one dataset derived from cell lines treated with NRF2-activating isothiocyanate, SFN, or have constitutively active NRF2 [33, 38, 42]. This is in comparison to the background rate of 13.7%, where a total of 3553 genes contained NRF2-ChIP bound regions within 10 Kb of the TSS of the approximately 26,000 identified transcribed genes in the human genome (hg38). Therefore, the NRF2 biomarker genes are more significantly represented with NRF2-bound regions (Fisher’s Exact test, p<0.05).”**
